# (1*E*,4*E*)-1,5-Bis(2,4-dimethyl­phen­yl)penta-1,4-dien-3-one

**DOI:** 10.1107/S1600536809033583

**Published:** 2009-08-29

**Authors:** Zhiguo Feng, Junhua Li, Yi Lin

**Affiliations:** a School of Life Science, Anhui Agricultural University, 130 West Yangtze Road, Hefei, Anhui Province 230036, People’s Republic of China; b Shenzhen Wanxin Pharmatech Co Ltd, 1-108 Bioincubator building, 1st Gaoxin Road, Shenzhen, Guangdong 518057, People’s Republic of China

## Abstract

In the title compound, C_21_H_22_O, a derivative of the biologically active compound curcumin, the dihedral angle between the aromatic ring planes is 20.57 (11)°.

## Related literature

For backgound to cucucmin and its biological properties, see: Began *et al.* (1999 ▶); Gautam *et al.* (2007 ▶); Liang *et al.* (2008 ▶); Liang, Shao *et al.* (2009 ▶); Liang, Tian *et al.* (2007 ▶); Liang, Yang *et al.* (2007 ▶); Liang, Zhou *et al.* (2009 ▶); Maheshwari *et al.* (2006 ▶); Zhao *et al.* (2009 ▶).
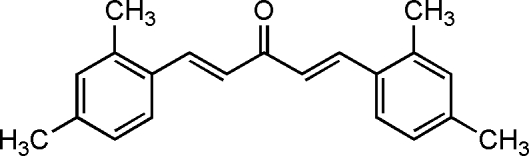


## Experimental

### 

#### Crystal data

C_21_H_22_O
*M* *_r_* = 290.39
Monoclinic, 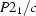

*a* = 4.9548 (7) Å
*b* = 26.555 (4) Å
*c* = 12.9632 (19) Å
β = 94.090 (3)°
*V* = 1701.3 (4) Å^3^
*Z* = 4
Mo *K*α radiation
μ = 0.07 mm^−1^
*T* = 293 K
0.42 × 0.37 × 0.26 mm

#### Data collection

Bruker SMART CCD diffractometer
Absorption correction: multi-scan (*SADABS*; Bruker, 2002 ▶) *T*_min_ = 0.769, *T*_max_ = 1.000
8842 measured reflections
3128 independent reflections
2007 reflections with *I* > 2σ(*I*)
*R*_int_ = 0.095

#### Refinement

*R*[*F* ^2^ > 2σ(*F* ^2^)] = 0.065
*wR*(*F* ^2^) = 0.173
*S* = 0.97
3128 reflections
203 parameters
H-atom parameters constrained
Δρ_max_ = 0.21 e Å^−3^
Δρ_min_ = −0.21 e Å^−3^

### 

Data collection: *SMART* (Bruker, 2002 ▶); cell refinement: *SAINT* (Bruker, 2002 ▶); data reduction: *SAINT*; program(s) used to solve structure: *SHELXS97* (Sheldrick, 2008 ▶); program(s) used to refine structure: *SHELXL97* (Sheldrick, 2008 ▶); molecular graphics: *SHELXTL* (Sheldrick, 2008 ▶); software used to prepare material for publication: *SHELXTL*.

## Supplementary Material

Crystal structure: contains datablocks I, global. DOI: 10.1107/S1600536809033583/hb5050sup1.cif

Structure factors: contains datablocks I. DOI: 10.1107/S1600536809033583/hb5050Isup2.hkl

Additional supplementary materials:  crystallographic information; 3D view; checkCIF report

## Figures and Tables

**Fig. 1.**
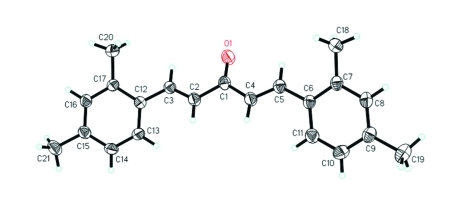
The molecular structure of (I) shoiwng 50% displacement ellipsoids for the non-hydrogen atoms.

## supplementary crystallographic information

### Comment

The title compound, C_21_H_22_O, (1E,4E)-1,5-bis(2,4-dimethylphenyl)penta-1,4-dien-3-one (I), is a mono-carbonyl analogue of curcumin. Curcumin has been found to possess a variety of pharmaceutical applications, for example, inhibiting the mutations and the formation of tumors, antioxidation, anti-inflammation and anti-virus (Began *et al.*, 1999; Maheshwari *et al.*, 2006; Gautam *et al.*, 2007). According to the structural disadvantages of curcumin which is considered to be responsible for its weak pharmacokinetic profiles, a series of mono-carbonyl analogues have been designed and synthesized, and their biological activity *in vitro* and *in vivo* were evaluated.

Derivatives of dibenzylidene acetone, cyclopentanone and cyclohexanone exhibit potent anti-inflammatory, antibacterial and anti-cancer activity.(Liang *et al.*, 2008; Liang *et al.*, 2008; Liang, Shao *et al.*, 2009; Liang, Zhou *et al.*, 2009). This fact leads to the significance of these synthetic mono-carbonyl analogues of curcumin. Several of these derivatives have been reported their crystal structures (Liang, Tian *et al.*, 2007; Liang, Yang *et al.*, 2007; Zhao *et al.*, 2009). In the present paper, we describe the crystal structure of the title compound C_21_H_22_O (I) here. Its geometrical parameters of are normal, the dihedral angle between the six-membered aromatic ring planes is 20.57 (11)°.

### Experimental

To a solution of 15 mmol 2,4-dimethylbenzaldehyde in MeOH (10 ml) was added 7.5 mmol acetone. The solution was stirred at room temperature for 20 min, followed by added dropwise 20% (*w*/*v*) NaOH (1.5 ml, 7.5 mmol). The mixture was stirred at RT and monitored with TLC. When the reaction finished, the residue was poured into saturated NH_4_Cl solution and filtered. The precipitate was washed and purified by chromatography over silica gel using CH_2_Cl_2_ / CH_3_OH as the eluent to afford the pure product (yield: 38.2%). Colourless blokcs of (I) were grown in a CH_2_Cl_2_—CH_3_OH mixture (6:2 *v*/*v*) by slow evaporation (mp 436–437 K). 1*H*-NMR (CDCl_3_): 2.34 (s, 6H, Ar_4_—CH_3_), 2.44 (s, 6H, Ar_2_—CH_3_), 6.96 (d, 2H, J=16.0 Hz, =CH—C=O), 7.05 (m, 4H, Ar—H_3_,_5_), 7.56 (d, 2H, J=8.0 Hz, Ar—H_6_), 8.0 (d, 2H, J=16.0 Hz, Ar—CH=C). ESI-MS m/*z*: 603.8 (2*M*+Na)+, calcd for C_21_H_22_O: 290.4.

### Refinement

The H atoms were positioned geometrically (C—H = 0.93–0.97 Å) and refined as riding with *U*_iso_(H) = 1.2*U*_eq_(C) or 1.5*U*_eq_(methyl C).

### Crystal data

**Table:**

C_21_H_22_O	*F*(000) = 624
*M**_r_* = 290.39	*D*_x_ = 1.134 Mg m^−^^3^
Monoclinic, *P*2_1_/*c*	Mo *K*α radiation, λ = 0.71073 Å
Hall symbol: -P 2ybc	Cell parameters from 1845 reflections
*a* = 4.9548 (7) Å	θ = 4.4–44.1°
*b* = 26.555 (4) Å	µ = 0.07 mm^−^^1^
*c* = 12.9632 (19) Å	*T* = 293 K
β = 94.090 (3)°	Prism, green
*V* = 1701.3 (4) Å^3^	0.42 × 0.37 × 0.26 mm
*Z* = 4	

### Data collection

**Table:**

Bruker SMART CCD diffractometer	3128 independent reflections
Radiation source: fine-focus sealed tube	2007 reflections with *I* > 2σ(*I*)
graphite	*R*_int_ = 0.095
ω scans	θ_max_ = 25.5°, θ_min_ = 1.5°
Absorption correction: multi-scan (*SADABS*; Bruker, 2002)	*h* = −5→6
*T*_min_ = 0.769, *T*_max_ = 1.000	*k* = −31→32
8842 measured reflections	*l* = −7→15

### Refinement

**Table:**

Refinement on *F*^2^	Primary atom site location: structure-invariant direct methods
Least-squares matrix: full	Secondary atom site location: difference Fourier map
*R*[*F*^2^ > 2σ(*F*^2^)] = 0.065	Hydrogen site location: inferred from neighbouring sites
*wR*(*F*^2^) = 0.173	H-atom parameters constrained
*S* = 0.97	*w* = 1/[σ^2^(*F*_o_^2^) + (0.0858*P*)^2^] where *P* = (*F*_o_^2^ + 2*F*_c_^2^)/3
3128 reflections	(Δ/σ)_max_ = 0.057
203 parameters	Δρ_max_ = 0.21 e Å^−^^3^
0 restraints	Δρ_min_ = −0.21 e Å^−^^3^

### Special details

**Table:**

Geometry. All e.s.d.'s (except the e.s.d. in the dihedral angle between two l.s. planes) are estimated using the full covariance matrix. The cell e.s.d.'s are taken into account individually in the estimation of e.s.d.'s in distances, angles and torsion angles; correlations between e.s.d.'s in cell parameters are only used when they are defined by crystal symmetry. An approximate (isotropic) treatment of cell e.s.d.'s is used for estimating e.s.d.'s involving l.s. planes.
Refinement. Refinement of *F*^2^ against ALL reflections. The weighted *R*-factor *wR* and goodness of fit *S* are based on *F*^2^, conventional *R*-factors *R* are based on *F*, with *F* set to zero for negative *F*^2^. The threshold expression of *F*^2^ > σ(*F*^2^) is used only for calculating *R*-factors(gt) *etc*. and is not relevant to the choice of reflections for refinement. *R*-factors based on *F*^2^ are statistically about twice as large as those based on *F*, and *R*- factors based on ALL data will be even larger.

### Fractional atomic coordinates and isotropic or equivalent isotropic displacement parameters (Å^2^)

**Table:**

	*x*	*y*	*z*	*U*_iso_*/*U*_eq_	
O1	1.4353 (4)	0.35616 (7)	0.08357 (12)	0.0700 (5)	
C1	1.2047 (5)	0.36963 (8)	0.09662 (16)	0.0510 (6)	
C2	1.0758 (5)	0.35782 (8)	0.19171 (16)	0.0528 (6)	
H2	0.9031	0.3703	0.1994	0.063*	
C3	1.1920 (4)	0.33037 (8)	0.26714 (16)	0.0479 (6)	
H3	1.3621	0.3173	0.2566	0.057*	
C4	1.0435 (5)	0.39838 (8)	0.01739 (16)	0.0545 (6)	
H4	0.8698	0.4083	0.0313	0.065*	
C5	1.1329 (5)	0.41070 (8)	−0.07216 (16)	0.0516 (6)	
H5	1.3019	0.3982	−0.0860	0.062*	
C6	0.9942 (4)	0.44212 (7)	−0.15263 (16)	0.0473 (6)	
C7	1.0565 (4)	0.43873 (7)	−0.25633 (16)	0.0493 (6)	
C8	0.9237 (5)	0.47084 (8)	−0.32771 (17)	0.0557 (6)	
H8	0.9633	0.4684	−0.3966	0.067*	
C9	0.7367 (5)	0.50614 (8)	−0.30192 (18)	0.0570 (6)	
C10	0.6759 (5)	0.50852 (9)	−0.19985 (19)	0.0645 (7)	
H10	0.5482	0.5316	−0.1801	0.077*	
C11	0.8023 (5)	0.47709 (9)	−0.12683 (18)	0.0603 (6)	
H11	0.7577	0.4794	−0.0585	0.072*	
C12	1.0812 (4)	0.31833 (7)	0.36552 (15)	0.0455 (5)	
C13	0.8956 (5)	0.34978 (8)	0.40688 (17)	0.0564 (6)	
H13	0.8429	0.3789	0.3710	0.068*	
C14	0.7855 (5)	0.33986 (9)	0.49876 (18)	0.0614 (7)	
H14	0.6607	0.3620	0.5239	0.074*	
C15	0.8597 (5)	0.29692 (9)	0.55428 (16)	0.0544 (6)	
C16	1.0474 (4)	0.26532 (9)	0.51378 (16)	0.0535 (6)	
H16	1.0998	0.2365	0.5506	0.064*	
C17	1.1613 (4)	0.27453 (8)	0.42090 (15)	0.0466 (5)	
C18	1.2581 (5)	0.40116 (8)	−0.29188 (17)	0.0626 (7)	
H18A	1.2595	0.4025	−0.3658	0.094*	
H18B	1.2081	0.3679	−0.2712	0.094*	
H18C	1.4350	0.4091	−0.2613	0.094*	
C19	0.5986 (6)	0.54084 (9)	−0.3819 (2)	0.0795 (8)	
H19A	0.6863	0.5383	−0.4453	0.119*	
H19B	0.6098	0.5749	−0.3572	0.119*	
H19C	0.4120	0.5313	−0.3938	0.119*	
C20	1.3587 (5)	0.23772 (9)	0.38199 (18)	0.0640 (7)	
H20A	1.3890	0.2110	0.4313	0.096*	
H20B	1.5267	0.2545	0.3725	0.096*	
H20C	1.2871	0.2240	0.3172	0.096*	
C21	0.7377 (5)	0.28438 (12)	0.65406 (18)	0.0784 (8)	
H21A	0.6361	0.3127	0.6761	0.118*	
H21B	0.8792	0.2765	0.7060	0.118*	
H21C	0.6198	0.2559	0.6438	0.118*	

### Atomic displacement parameters (Å^2^)

**Table:**

	*U*^11^	*U*^22^	*U*^33^	*U*^12^	*U*^13^	*U*^23^
O1	0.0565 (11)	0.0924 (13)	0.0619 (11)	0.0062 (9)	0.0100 (9)	0.0172 (9)
C1	0.0505 (14)	0.0541 (13)	0.0482 (13)	−0.0077 (11)	0.0023 (11)	0.0032 (10)
C2	0.0491 (13)	0.0618 (14)	0.0476 (13)	−0.0010 (11)	0.0044 (11)	0.0058 (11)
C3	0.0476 (13)	0.0479 (12)	0.0482 (13)	−0.0034 (10)	0.0043 (10)	−0.0009 (10)
C4	0.0522 (14)	0.0625 (14)	0.0492 (13)	0.0007 (11)	0.0061 (11)	0.0072 (11)
C5	0.0558 (14)	0.0491 (13)	0.0498 (13)	−0.0050 (10)	0.0036 (11)	−0.0002 (10)
C6	0.0546 (14)	0.0411 (11)	0.0459 (13)	−0.0053 (10)	0.0016 (10)	−0.0001 (9)
C7	0.0548 (13)	0.0456 (12)	0.0474 (13)	−0.0087 (10)	0.0037 (11)	0.0022 (10)
C8	0.0668 (16)	0.0528 (13)	0.0474 (13)	−0.0088 (12)	0.0034 (11)	0.0045 (11)
C9	0.0682 (16)	0.0427 (12)	0.0588 (15)	−0.0048 (12)	−0.0049 (12)	0.0009 (11)
C10	0.0678 (17)	0.0532 (14)	0.0716 (17)	0.0092 (12)	−0.0008 (13)	−0.0069 (13)
C11	0.0715 (17)	0.0579 (14)	0.0520 (14)	−0.0001 (12)	0.0084 (12)	−0.0018 (11)
C12	0.0447 (12)	0.0481 (12)	0.0435 (12)	−0.0012 (10)	0.0020 (10)	−0.0009 (10)
C13	0.0607 (15)	0.0549 (13)	0.0539 (14)	0.0141 (11)	0.0074 (12)	0.0058 (11)
C14	0.0598 (15)	0.0696 (16)	0.0564 (15)	0.0172 (13)	0.0156 (12)	0.0002 (12)
C15	0.0517 (14)	0.0657 (15)	0.0461 (13)	0.0025 (12)	0.0056 (11)	0.0027 (11)
C16	0.0531 (14)	0.0586 (14)	0.0487 (13)	0.0035 (11)	0.0031 (11)	0.0115 (11)
C17	0.0455 (12)	0.0462 (12)	0.0479 (13)	0.0023 (10)	0.0015 (10)	0.0012 (10)
C18	0.0700 (16)	0.0639 (15)	0.0549 (14)	0.0062 (13)	0.0110 (12)	−0.0017 (12)
C19	0.095 (2)	0.0616 (16)	0.0795 (19)	0.0047 (14)	−0.0140 (16)	0.0103 (14)
C20	0.0720 (17)	0.0580 (14)	0.0632 (15)	0.0108 (13)	0.0140 (13)	0.0003 (12)
C21	0.0716 (18)	0.107 (2)	0.0586 (16)	0.0085 (16)	0.0199 (13)	0.0149 (15)

### Geometric parameters (Å, °)

**Table:**

O1—C1	1.221 (3)	C12—C17	1.409 (3)
C1—C2	1.462 (3)	C13—C14	1.370 (3)
C1—C4	1.469 (3)	C13—H13	0.9300
C2—C3	1.319 (3)	C14—C15	1.384 (3)
C2—H2	0.9300	C14—H14	0.9300
C3—C12	1.459 (3)	C15—C16	1.383 (3)
C3—H3	0.9300	C15—C21	1.504 (3)
C4—C5	1.313 (3)	C16—C17	1.388 (3)
C4—H4	0.9300	C16—H16	0.9300
C5—C6	1.468 (3)	C17—C20	1.496 (3)
C5—H5	0.9300	C18—H18A	0.9600
C6—C11	1.387 (3)	C18—H18B	0.9600
C6—C7	1.403 (3)	C18—H18C	0.9600
C7—C8	1.389 (3)	C19—H19A	0.9600
C7—C18	1.507 (3)	C19—H19B	0.9600
C8—C9	1.376 (3)	C19—H19C	0.9600
C8—H8	0.9300	C20—H20A	0.9600
C9—C10	1.379 (3)	C20—H20B	0.9600
C9—C19	1.513 (3)	C20—H20C	0.9600
C10—C11	1.379 (3)	C21—H21A	0.9601
C10—H10	0.9300	C21—H21B	0.9601
C11—H11	0.9300	C21—H21C	0.9601
C12—C13	1.379 (3)		
			
O1—C1—C2	121.6 (2)	C12—C13—H13	118.7
O1—C1—C4	121.5 (2)	C13—C14—C15	120.2 (2)
C2—C1—C4	116.9 (2)	C13—C14—H14	119.9
C3—C2—C1	123.4 (2)	C15—C14—H14	119.9
C3—C2—H2	118.3	C14—C15—C16	117.6 (2)
C1—C2—H2	118.3	C14—C15—C21	121.4 (2)
C2—C3—C12	126.7 (2)	C16—C15—C21	121.0 (2)
C2—C3—H3	116.6	C15—C16—C17	123.3 (2)
C12—C3—H3	116.7	C15—C16—H16	118.4
C5—C4—C1	123.1 (2)	C17—C16—H16	118.4
C5—C4—H4	118.4	C16—C17—C12	118.1 (2)
C1—C4—H4	118.4	C16—C17—C20	119.59 (19)
C4—C5—C6	126.8 (2)	C12—C17—C20	122.3 (2)
C4—C5—H5	116.6	C7—C18—H18A	109.5
C6—C5—H5	116.6	C7—C18—H18B	109.5
C11—C6—C7	118.4 (2)	H18A—C18—H18B	109.5
C11—C6—C5	120.2 (2)	C7—C18—H18C	109.5
C7—C6—C5	121.4 (2)	H18A—C18—H18C	109.5
C8—C7—C6	118.2 (2)	H18B—C18—H18C	109.5
C8—C7—C18	119.7 (2)	C9—C19—H19A	109.5
C6—C7—C18	122.09 (19)	C9—C19—H19B	109.5
C9—C8—C7	123.5 (2)	H19A—C19—H19B	109.5
C9—C8—H8	118.3	C9—C19—H19C	109.5
C7—C8—H8	118.3	H19A—C19—H19C	109.5
C8—C9—C10	117.5 (2)	H19B—C19—H19C	109.5
C8—C9—C19	121.9 (2)	C17—C20—H20A	109.5
C10—C9—C19	120.7 (2)	C17—C20—H20B	109.5
C11—C10—C9	120.8 (2)	H20A—C20—H20B	109.5
C11—C10—H10	119.6	C17—C20—H20C	109.5
C9—C10—H10	119.6	H20A—C20—H20C	109.5
C10—C11—C6	121.7 (2)	H20B—C20—H20C	109.5
C10—C11—H11	119.2	C15—C21—H21A	109.5
C6—C11—H11	119.1	C15—C21—H21B	109.5
C13—C12—C17	118.2 (2)	H21A—C21—H21B	109.5
C13—C12—C3	120.67 (19)	C15—C21—H21C	109.5
C17—C12—C3	121.08 (19)	H21A—C21—H21C	109.5
C14—C13—C12	122.6 (2)	H21B—C21—H21C	109.5
C14—C13—H13	118.7		
			
O1—C1—C2—C3	−3.1 (3)	C9—C10—C11—C6	0.1 (4)
C4—C1—C2—C3	176.8 (2)	C7—C6—C11—C10	−0.8 (3)
C1—C2—C3—C12	177.8 (2)	C5—C6—C11—C10	177.5 (2)
O1—C1—C4—C5	1.1 (4)	C2—C3—C12—C13	−26.1 (3)
C2—C1—C4—C5	−178.8 (2)	C2—C3—C12—C17	154.2 (2)
C1—C4—C5—C6	−175.49 (19)	C17—C12—C13—C14	−0.5 (3)
C4—C5—C6—C11	24.6 (3)	C3—C12—C13—C14	179.7 (2)
C4—C5—C6—C7	−157.2 (2)	C12—C13—C14—C15	0.1 (4)
C11—C6—C7—C8	0.4 (3)	C13—C14—C15—C16	0.4 (4)
C5—C6—C7—C8	−177.83 (18)	C13—C14—C15—C21	−178.5 (2)
C11—C6—C7—C18	−178.7 (2)	C14—C15—C16—C17	−0.4 (3)
C5—C6—C7—C18	3.0 (3)	C21—C15—C16—C17	178.4 (2)
C6—C7—C8—C9	0.7 (3)	C15—C16—C17—C12	0.1 (3)
C18—C7—C8—C9	179.9 (2)	C15—C16—C17—C20	−178.8 (2)
C7—C8—C9—C10	−1.4 (3)	C13—C12—C17—C16	0.4 (3)
C7—C8—C9—C19	179.3 (2)	C3—C12—C17—C16	−179.83 (19)
C8—C9—C10—C11	1.0 (4)	C13—C12—C17—C20	179.2 (2)
C19—C9—C10—C11	−179.7 (2)	C3—C12—C17—C20	−1.0 (3)
